# Potentially Functional Polymorphisms in the *CASP7* Gene Contribute to Gastric Adenocarcinoma Susceptibility in an Eastern Chinese Population

**DOI:** 10.1371/journal.pone.0074041

**Published:** 2013-09-10

**Authors:** Meng-Yun Wang, Mei-Ling Zhu, Jing He, Ting-Yan Shi, Qiao-Xin Li, Ya-Nong Wang, Jin Li, Xiao-Yan Zhou, Meng-Hong Sun, Xiao-Feng Wang, Ya-Jun Yang, Jiu-Cun Wang, Li Jin, Qing-Yi Wei

**Affiliations:** 1 Cancer Institute, Fudan University Shanghai Cancer Center, Shanghai, China; 2 Department of Oncology, Shanghai Medical College, Fudan University, Shanghai, China; 3 Department of Abdominal Surgery, Fudan University Shanghai Cancer Center, Shanghai, China; 4 Department of Medical Oncology, Fudan University Shanghai Cancer Center, Shanghai, China; 5 Department of Pathology, Fudan University Shanghai Cancer Center, Shanghai, China; 6 Ministry of Education Key Laboratory of Contemporary Anthropology, State Key Laboratory of Genetic Engineering, School of Life Sciences, Fudan University, Shanghai, China; 7 Fudan-Taizhou Institute of Health Sciences, Taizhou, Jiangsu, China; 8 Department of Epidemiology, The University of Texas MD Anderson Cancer Center, Houston, Texas, United States of America; MOE Key Laboratory of Environment and Health, School of Public Health, Tongji Medical College, Huazhong University of Science and Technology, China

## Abstract

**Background:**

Caspase 7 (*CASP7*) is an important regulator and executioner in the apoptosis pathway and plays a crucial role in cancer development and progression. However, few studies have evaluated associations between functional single nucleotide polymorphisms (SNPs) in the 3′ untranslational region (UTR) of *CASP7* and risk of gastric cancer.

**Methods:**

In a case-control study of 1117 patients with gastric cancer and 1146 cancer-free controls with frequency matching on age and sex, we genotyped four potentially functional SNPs (rs4353229T>C, rs10787498T>G, rs1127687G>A and rs12247479G>A) located in the microRNA binding sites of the *CASP7* 3′ UTR by using Taqman assays and evaluated their associations with risk of gastric cancer by using logistic regression analyses as well as multifactorial dimension reduction (MDR) analysis.

**Results:**

In the single-locus analysis, only the *CASP7* rs4353229 TT genotype was associated with 0.83-fold decreased risk (95% confidence interval [CI] = 0.70–0.98) of gastric cancer under a recessive model, compared with the CT/CC genotypes. In the combined analysis of all four SNPs, we found that the risk of gastric cancer decreased by 19% in those carrying any of the risk genotypes (adjusted odds ratio = 0.81, 95% CI = 0.68–0.96), compared with those carrying zero risk genotypes, and this risk was more evident in subgroups of younger age (<59 years), females, non-smokers, non-drinkers and patients with non-gastric cardia adenocarcinoma. Further MDR analysis suggested some evidence of interactions between the combined genotypes and other risk factors for gastric cancer.

**Conclusions:**

Potentially functional *CASP7* variants may contribute to risk of gastric cancer. Larger studies with different ethnic populations are warranted to validate our findings.

## Introduction

Gastric cancer is one of the most common cancers worldwide and the second most frequent cause of cancer death, accounting for 8% of the total new cases and 10% of total deaths worldwide in 2008 [1], [2]. The highest incidence occurs in Eastern Asia (including China, Japan and Korea), Europe and South America (including Australia and New Zealand). Regions associated with low risk include North America, Africa, South Asia, and Oceania [2]. Strong environmental component contributes to the etiology of gastric cancer, including infection of *Helicobacter pylori* (*H. pylori*), a major risk factor for gastric cancer, which has been classified as a Group 1 carcinogen by the International Agency for Research on Cancer (IARC) and the World Health Organization (WHO) [3]. Other important environmental risk factors include smoking, high intake of various traditional salt-preserved foods or salt, and low consumption of fresh fruits and vegetables [4], [5]. The fact that only a small portion of individuals will develop gastric cancer under similar exposures suggests that genetic variations may also contribute to individual susceptibility to gastric cancer.

Apoptosis, also known as the programmed cell death, plays a crucial role in the regulation of cellular homeostasis in multicellular organisms [6]. It is well known that deregulation of apoptosis may facilitate the accumulation of somatic mutations and thereby contribute to cancer development [7]. Caspases (CASPs) are a family of cysteine-aspartic acid proteases that coordinate in cellular regulation and execution of apoptosis [8]. Based on their proapoptotic functions, CASPs can be divided into “initiators”, which transmit death signals, and “executioners”, which execute a coordinated program of proteolysis that leads to the final cell death program [8].

The *CASP7* gene, located in chromosome 10q25.1–10q25.2, is a well-known effector caspase that is critical for apoptosis induction. Therefore, altered functions of this gene, likely caused by genetic alterations such as mutations, or functional single nucleotide polymorphisms (SNPs) may lead to deregulated apoptosis. For example, *CASP7* has been proposed as a positional candidate for susceptibility to insulin-dependent diabetes mellitus (IDDM) and inactivating mutations of the *CASP7* gene might lead to the loss of its apoptotic function and contribute to the pathogenesis of some human solid cancers [9], [10]. Up to date, no published studies have investigated associations between *CASP7* SNPs and gastric cancer risk in Chinese populations, except for the overall risk assessment in genome-wide association studies [11].

MicroRNAs (miRNAs) are a class of small non-coding RNA molecules that regulate gene expression by binding to the 3′untranslated region (UTR) of their target mRNAs, resulting in mRNA cleavage or translation repression [12]. Sequence variations, such as SNPs, located in the 3′-UTR of miRNA target genes are also likely to either abolish or weaken a microRNA target or to create an imperfect sequence matched to the seed of a microRNA, thereby disrupting the microRNA–mRNA interaction and affecting the expression of microRNA targets [13]. There is evidence that SNPs in the miRNA binding sites may be associated with individual susceptibility to cancer [14], [15], [16]. However, few studies have investigated the role of SNPs at the miRNA binding sites of *CAPS*7 in the etiology of gastric cancer in Chinese populations.

Based on the important role that *CASP7* plays in carcinogenesis, we hypothesize that potentially functional SNPs, such as those located in the 3′-UTR of *CASP7,* may influence the *CASP7* expression, thereby modulating susceptibility to gastric cancer. To test this hypothesis, we used public databases to identify SNPs in the 3′-UTR of *CASP7*, which may potentially affect the miRNA binding and investigated their associations with risk of developing gastric cancer in an Eastern Chinese population.

## Materials and Methods

### Study Population

The subjects were recruited from an ongoing case-control study as described previously [17]. The present study included 1,117 unrelated ethnic Han Chinese patients with newly diagnosed and histopathologically confirmed primary gastric adenocarcinoma, who were recruited from Fudan University Shanghai Cancer Center between January 2009 and March 2011. All patients came from Eastern China, including Shanghai City, Jiangsu Province, Zhejiang Province and the surrounding regions. Participants who had gastric adenosquamous, squamous cell carcinoma, neuroendocrine tumor, stromal tumor, metastasized cancer from other organs or any histopathologic diagnosis other than gastric adenocarcinoma were excluded from this study. The selection criteria of controls included no history of cancer, who were frequency matched to the cases on sex and age (±5 years). At recruitment, a written informed consent was obtained from each subject before the in-person interview to obtain demographic data and environmental exposure history, including age, sex, ethnicity, smoking and alcohol consumption. After interview, each participant donated a sample of approximately 10-mL blood, of which 1 mL was used for genomic DNA extraction. The Institutional Review Board of Fudan University Shanghai Cancer Center approved this study.

### SNP Selection and Genotyping

**Figure 1:**
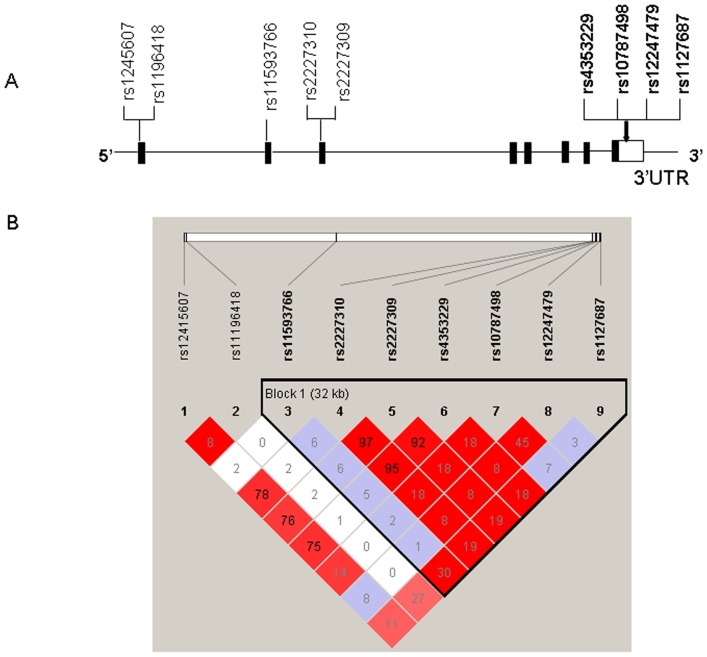
Gene map and linkage disequilibrium (LD) blocks of the *CASP7* gene. (A) Gene map and potentially functional polymorphisms in the *CASP7* gene predicted by SNPinfo. Exons are marked by black blocks, and 3′-UTRs by white blocks. SNPs studied in the present study were in bold. (B) Pairwise linkage disequilibrium (LD) among four selected SNPs of *CASP7*. The value within each diamond represents the pairwise correlation between SNPs (measured as r^2^) defined by the upper left and the upper right sides of the diamond. The red-to-white gradient reflects higher to lower LD values.

The potentially functional SNPs were selected from the NCBI dbSNP database (http://www.ncbi.nlm.nih.gov/projects/SNP) and the International HapMap Project database (http://hapmap.ncbi.nlm.nih.gov/) based on the following criteria: (1) located at the 3′-UTR of *CASP7* (**Figure 1A** **)**, (2) reported to be common SNPs: minor allele frequency (MAF) was at least 5% in Chinese populations, (3) with low linkage disequilibrium (LD) using an r^2^ threshold of <0.8 for each paired SNPs (**Figure 1B**), and (4) predicted as a potentially functional SNP that might be at an miRNA binding site by using the SNP function prediction (FuncPred) software (http://snpinfo.niehs.nih.gov/snpinfo/snpfunc.htm) and the TargetScan online tool. As a result, four potentially functional SNPs were selected for genotyping: (rs4353229T>C, rs10787498T>G, rs1127687G>A and rs12247479G>A).

We performed genomic DNA extraction and genotyping as previously described [18] with a successful genotyping rate of 97.5% by using the TaqMan probe assays (Applied Biosystems, Foster City, CA, USA) on a 7900 HT sequence detector system (Applied Biosystems) according to the manufacturer’s protocol. Genotyping of each sample was automatically attributed using the SDS 2.4 software for allelic discrimination. Ten percent of the samples chosen at random were genotyped again for quality control, and the discrepancy rate was less than 0.1%.

### Genotype and mRNA Expression Data of Lymphoblastoid Cell Lines from HapMap Database

To explore the functionality of the *CASP7* rs4353229 T>C SNP, we used publically available data on *CASP7* genotypes and transcript expression levels from 270 lymphoblastoid cell lines available online (http://app3.titan.uio.no/biotools/help.php?app=snpexp) [19] for the genotype–phenotype association analysis. The genotyping data were from the HapMap phase II release 23 data set consisting of 3.96 million SNP genotypes from 270 individuals from all population (45 CHB +45 JPT+90 CEU+90YRI) as previously described [20].

### Statistical Analysis

Two-sided chi-square tests were used to evaluate differences in the selected demographic variables of age, sex, smoking and drinking status between cases and controls. The deviation of genotype distribution from Hardy–Weinberg equilibrium was tested by a goodness-of-fit chi-square test to compare the expected genotype frequencies with observed genotype frequencies in cancer-free controls. The associations between *CASP7* genotypes and risk of gastric cancer were estimated by odds ratios (ORs) and their 95% confidence intervals (CIs), which were computed by using both univariate and multivariate logistic regression models. The associations were also evaluated in subgroup analysis by stratifying age, sex, smoking status, drinking status and tumor site. A homogeneity test was performed to compare the difference between genotype-related ORs of different subgroups.

Multifactor dimensionality reduction (MDR) analysis was performed to detect possible interactions, which was conducted by using the MDR V2.0 beta 8.2 program to further explore high-order gene-environment interactions that were individually involved in gastric cancer risk [21]. A single best model was considered, if it had minimal prediction error and maximal cross-validation consistency (CVC), which measures the number of times of 10 divisions of the data, on which the best model was built. Statistical significance was further evaluated by a 1000-time permutation test to compare observed testing accuracies with those expected under the null hypothesis of no association. Haplotype frequencies and individual haplotypes were estimated and analyzed by Statistical Analysis Software PROC HAPLOTYPE.

We also calculated the false-positive reporting probability (FPRP) for all significant results observed in the present study to detect the false-positive findings [22]. We assigned a prior probability of 0.1 to detect an OR of 1.56 (for a risk effect) or 0.67 (for a protective effect) for an association with genotypes of each SNP under investigation. Only significant results with an FPRP value less than 0.20 were considered a noteworthy association. All statistical analyses were performed by using Statistical Analysis Software (v.9.1 SAS Institute, Cary, NC), and all *P* values were two-sided with a 0.05 significance level.

## Results

### Characteristics of the Study Population

As shown in **Table 1**, the study population consisted of 1,117 gastric cancer patients and 1,146 controls with complete genotyping data in the final analysis. The cases and controls appeared to be adequately frequency-matched for age and sex (*P* = 0.321 and 0.479, respectively). The mean age was similar between cases (58.60±11.38 years) and controls (59.30±11.30 years). Nevertheless, there were more ever smokers and drinkers among the controls (49.7% and 28.5%, respectively) than among the cases (38.8% and 23.9%, respectively). Thereafter, these variables (i.e., age, sex, smoking status and drinking status) were further adjusted for in the subsequent multivariate logistic regression analyses. Of the cases, 303 (27.1%) had gastric cardia adenocarcinoma (GCA), and 814 (72.9%) had gastric non-cardia adenocarcinoma (NGCA).

**Table 1:** Frequency distributions of selected characteristics of gastric cancer cases and cancer-free controls.

Variables	Cases No. (%)	Controls No. (%)		P^a^
All subjects	1,117 (100.0)	1,146 (100.0)		
Age, yr				0.321
Range	21–86	22–86		
Mean^b^	58.6±11.38	59.3±11.30		
Age group				
≤50	234 (20.4)	233 (20.9)		
51–60	375 (32.7)	380 (34.0)		
61–70	370 (32.3)	335 (30.0)		
>70	167 (14.6)	169 (15.1)		
Sex				0.479
Males	793 (71.0)	798 (69.6)		
Females	324 (29.0)	348 (30.4)		
Drinking status				0.015
Ever	267 (23.9)		327 (28.5)	
Never	850 (76.1)		819 (71.5)	
Smoking status				<0.0001
Ever	433 (38.8)		570 (49.7)	
Never	684 (61.2)		576 (50.3)	
Pack-years				<0.0001
0	684 (61.2)		576 (50.3)	
≤25 (mean)	225 (20.1)		338 (29.5)	
>25 (mean)	208 (18.6)		232 (20.2)	
Tumor site				
GCA	303 (27.1)		–	
NGCA	814 (72.9)		–	

GCA, gastric cardia adenocarcinoma; NGCA, non-gastric cardia adenocarcinoma.

a Two-sided χ^2^ test for distributions between cases and controls.

b Data were presented as mean ± SD.

### Associations between *CASP7* Genotypes and Risk of Gastric Cancer

The genotype distributions of the four potentially functional SNPs among the cases and controls and their associations with gastric cancer risk are summarized in **Table 2**. The genotype frequencies among the controls were in agreement with the Hardy-Weinberg equilibrium (all *P*>0.05). When we assessed the association of each individual SNP with gastric cancer risk under recessive models for genetic susceptibility, only the rs4353229 TT variant genotype was found to be associated with a significantly decreased risk of gastric cancer, with an adjusted OR of 0.83 (95% CI = 0.70–0.98) after adjustment for age, sex, smoking and drinking status. This significant OR was not observed for other three SNPs, although the ORs associated with their variant homozygous genotypes were towards a similar but not statistically significant [adjusted OR = 0.75, 95% CI = 0.50–1.12 for rs10787498 GG, 0.89 (0.62–1.29) for rs1127687AA, and 0.87 (0.49–1.55) for rs12247479AA].

**Table 2:** Logistic regression analysis of associations between the genotypes of *CASP7* and gastric cancer risk.

Variants	Genotypes	Cases (N = 1,117)	Controls (N = 1,146)	*P* a		Crude OR (95% CI)	*P*	Adjusted OR (95% CI)	*P* b
rs10787498									
	TT	727 (65.1)	734 (64.1)		0.286^c^	1.00		1.00	
	GT	347 (31.1)	352 (30.7)			1.00 (0.83–1.19)	0.959	1.00 (0.83–1.19)	0.960
	GG	43 (3.9)	60 (5.2)			0.72 (0.48–1.09)	0.117	0.74 (0.50–1.12)	0.154
	TT/GT	1074 (96.2)	1086 (94.8)			1.00		1.00	
	GG	43 (3.9)	60 (5.2)		0.114^d^	0.73 (0.49–1.08)	0.115	0.75 (0.50–1.12)	0.152
rs1127687									
	GG	684 (61.2)	702 (61.3)		0.769^c^	1.00		1.00	
	AG	376 (33.7)	378 (33.0)			1.02 (0.86–1.22)	0.819	1.02 (0.85–1.21)	0.862
	AA	57 (5.1)	66 (5.8)			0.89 (0.61–1.28)	0.523	0.90 (0.62–1.30)	0.575
	GG/AG	1060 (94.9)	1080 (94.2)			1.00		1.00	
	AA	57 (5.1)	66 (5.8)		0.491^d^	0.88 (0.61–1.27)	0.492	0.89 (0.62–1.29)	0.550
rs12247479									
	GG	885 (79.2)	889 (77.6)		0.615^c^	1.00		1.00	
	AG	210 (18.8)	231 (20.2)			0.91 (0.74–1.13)	0.394	0.91 (0.74–1.12)	0.380
	AA	22 (2.0)	26 (2.3)			0.85 (0.48–1.51)	0.581	0.86 (0.48–1.53)	0.601
	GG/AG	1095 (98.0)	1120 (97.7)			1.00		1.00	
	AA	22 (2.0)	26 (2.3)		0.621^d^	0.87 (0.49–1.54)	0.623	0.87 (0.49–1.55)	0.645
rs4353229									
	CC	197 (17.6)	189 (16.5)		0.074^c^	1.00		1.00	
	CT	535 (47.9)	509 (44.4)			1.01 (0.80–1.27)	0.944	1.00 (0.79–1.27)	0.978
	TT	385 (34.5)	448 (39.1)			0.83 (0.65–1.05)	0.118	0.83 (0.65–1.06)	0.135
	CC/CT	732 (65.5)	698 (60.9)			1.00		1.00	
	TT	385 (34.5)	448 (39.1)		**0.023** d	**0.82 (0.69–0.97)**	**0.023**	**0.83 (0.70–0.98)**	**0.032**
Combined effect of risk genotypes									
	0	732 (65.5)	692 (60.4)		**0.011**	1.00		1.00	
	≥1	385 (34.5)	454 (39.6)			**0.81 (0.68–0.95)**	**0.011**	**0.81 (0.68–0.96)**	**0.018**

CI, confidence interval; OR, odds ratio.

a Chi square test for genotype distributions between cases and controls.

b Adjusted for age, sex, smoking and drinking status in logistic regress models.

c For additive genetic models;

d for recessive genetic models. The results were in bold, if the 95% CI excluded 1 or *P*<0.05.

As shown in **Figure 1**, the four selected *CASP7* SNPs are not in high LD, but the effects of their homozygous variant genotypes on risk of gastric cancer tended to be protective in the same direction (**Table 2**) under recessive genetic models. Considering a possible joint effect for these four SNPs on risk of gastric cancer, we combined all the putative risk genotypes (i.e., rs10787498 GG, rs1127687 AA, rs12247479 AA and rs4353229 TT) to analyze their possible combined effect on risk of gastric cancer. When the “0” risk genotype group was used as the reference, the decreased risk of gastric cancer was 0.81 fold (95% CI = 0.68–0.96) for those who carried “1–4” risk genotypes. Although the number of subjects carrying “≥1” risk genotypes was almost identical to that for those carrying rs4353229 TT, the risk estimate did improve somewhat. Therefore, we also used this combined genotype group for further stratified analysis.

### Stratification Analysis

We further performed a stratification analysis of the associations of rs4353229 T>C and the combined genotypes of four SNPs with risk of gastric cancer by subgroups of age, sex, smoking and drinking status as well as tumor site, assuming a recessive model. As shown in **Table 3**, the rs4353229 homozygous TT variant genotype was associated with a significantly reduced gastric cancer risk in subgroups of younger subjects (defined as subjects ≤59 years old, adjusted OR = 0.77, 95% CI = 0.60–0.98), non-smokers (adjusted OR = 0.78, 95% CI = 0.62–0.98), non-drinkers (adjusted OR = 0.81, 95% CI = 0.66–0.99) and subjects with non-cardia cancer (adjusted OR = 0.81, 95% CI = 0.67–0.98). Likewise, similar results were found for those carrying “≥1” risk genotypes of the four SNPs. However, homogeneity tests suggested that there was no difference in risk estimates between strata, nor any evidence of gene-environment interactions between the variant genotypes and other selected variables on risk of gastric cancer (data not shown).

**Table 3:** Stratification analysis for associations between variant genotypes and gastric cancer risk.

Variables	rs4353229							Combined risk genotypes						
	(cases/controls)		Crude OR (95% CI)	*P*	Adjusted OR(95% CI)	*P* a	*P*_hom_	(cases/controls)		Crude OR(95% CI)	*P*	Adjusted OR^a^ (95% CI)	*P* a	*P*_hom_
	CT/CC	TT						0	≥1					
Age														
≤59	387/347	188/219	**0.77 (0.60–0.98)**	**0.035**	**0.77 (0.60–0.98)**	**0.033**	0.462	387/343	188/223	**0.75 (0.59–0.95)**	**0.019**	**0.75 (0.59–0.95)**	**0.018**	0.410
>59	345/351	197/229	0.88 (0.69–1.12)	0.280	0.90 (0.70–1.15)	0.391		345/349	197/231	0.86 (0.68–1.10)	0.219	0.89 (0.70–1.14)	0.345	
Sex														
Females	214/206	110/142	0.75 (0.55–1.02)	0.067	0.75 (0.55–1.03)	0.072	0.480	214/203	110/145	**0.72 (0.53–0.99)**	**0.040**	**0.73 (0.53–1.00)**	**0.047**	0.417
Males	518/492	275/306	0.85 (0.70–1.05)	0.129	0.86 (0.70–1.06)	0.158		518/489	275/309	0.84 (0.69–1.03)	0.095	0.85 (0.69–1.04)	0.120	
Smoking status														
Never	457/350	227/226	**0.77 (0.61–0.91)**	**0.026**	**0.78 (0.62–0.98)**	**0.035**	0.372	457/348	227/228	**0.76 (0.60–0.96)**	**0.019**	**0.77 (0.61–0.97)**	**0.026**	0.418
Ever	275/348	158/222	0.90 (0.70–1.17)	0.427	0.90 (0.70–1.17)	0.443		275/344	158/226	0.88 (0.98–1.13)	0.308	0.88 (0.68–1.14)	0.327	
Drinking status														
Never	563/500	287/319	**0.80 (0.65–0.98)**	**0.028**	**0.81 (0.66–0.99)**	**0.039**	0.586	563/496	287/323	**0.78 (0.64–0.96)**	**0.016**	**0.80 (0.65–0.97)**	**0.025**	0.604
Ever	169/198	98/129	0.89 (0.64–1.24)	0.493	0.89 (0.64–1.24)	0.489		169/196	98/131	0.87 (0.62–1.21)	0.403	0.87 (0.62–1.21)	0.398	
Site														
Cardia	194/698	109/448	0.88 (0.67–1.14)	0.321	0.88 (0.68–1.15)	0.361	0.581	194/692	109/454	0.86 (0.66–1.11)	0.248	0.87 (0.67–1.13)	0.300	0.581
Non–cardia	538/698	276/448	**0.80 (0.66–0.96)**	**0.019**	**0.81 (0.67–0.98)**	**0.029**		538/692	276/454	**0.78 (0.65–0.94)**	**0.010**	**0.79 (0.66–0.96)**	**0.016**	

CI, confidence interval; OR, odds ratio.

a Obtained in logistic regression models with adjustment for age, sex, smoking status and drinking status. *P*_hom_ derived from the homogeneity test. The results were in bold, if the 95% CI excluded 1 or *P*<0.05.

We further performed the MDR analysis to evaluate the interactions of all possible genotypes and their combinations of the four SNPs with other risk factors. As shown in **Table 4**, we found that the overall best MDR model was the eight factors model that had a maximum CVC (100/100) and a minimum prediction error (41.6%), which presents a better prediction than any one of the eight factors.

**Table 4:** MDR analysis for the risk of gastric cancer prediction with and without *CASP7* genotypes.

Best interaction models	Cross-validation	Average prediction error	*P* a
1	100/100	44.6%	<.0001
1,2	84/100	44.4%	<.0001
1,3,4	87/100	43.4%	<.0001
1,3,4,5	96/100	43.2%	<.0001
1,3,4,5,6	100/100	42.6%	<.0001
1,3,4,5,6,7	96/100	42.3%	<.0001
1,2,3,4,5,6,8	99/100	41.9%	<.0001
**1, 2, 3, 4, 5, 6, 7, 8**	**100/100**	**41.6%**	**<.0001**

MDR, multifactor dimensionality reduction.

a *P* value for 1000-fold permutation test. The best model with maximum cross-validation consistency and minimum prediction error rate was in bold. Labels: 1, smoking status; 2, rs12247479; 3, sex; 4, rs4353229; 5, age; 6, drink status; 7, rs1127687; 8,#rs10787498.

By using the SAS PROC HAPLOTYPE program, we inferred all the possible haplotypes based on the observed genotype data, of which four common (>10%) haplotypes (TGGC, TAGT, TGGT and GGAT) represented 92.1% of all haplotypes for the cases and 89.8% for the controls (**Table 5**). When the most common haplotype TGGC was used as the reference, none of the haplotypes were associated with a significant decreased risk of gastric cancer in logistic regression models, either in a univariate model or a multivariate model with adjustment for age, sex, smoking and alcohol use.

**Table 5:** Haplotype analysis for genotypes of *CASP7* and Gastric Cancer risk.

Haplotypes^a^	Haplotype frequencies				Crude OR (95% CI)	*P*	Adjusted OR(95% CI)	*P* a
	Cases		Controls					
	n	%	n	%				
T-G-G-C	927	41.6	867	37.9	1.00		1.00	
T-A-G-T	488	21.9	490	21.4	0.97 (0.83–1.13)	0.712	0.98 (0.83–1.14)	0.751
T-G-G-T	384	17.2	432	18.9	0.88 (0.74–1.02)	0.090	0.87 (0.74–1.03)	0.110
G-G-A-T	254	11.4	264	11.6	0.94 (0.77–1.14)	0.522	0.94 (0.78–1.15)	0.556

a Obtained in logistic regression models with adjustment for age, sex, smoking status and drinking status.

Finally, we calculated the FPRP values at different prior probability levels for all the observed significant associations (**Table 6**). For a prior probability of 0.1, assuming that the OR for specific genotype was 0.67 (protection), with statistical power of 0.997 and 0.964, the FPRP values were 0.172 and 0.151, respectively, for an association of the TT vs. CC/CT genotypes with a significantly reduced risk of gastric cancer in all individuals and subgroup of non-cardia. We also found them noteworthy for significant associations with reduced risk of gastric cancer for younger subjects (≤59 years), non-smokers, non-drinkers and non-cardia cancer among those with ≥1 risk genotypes, because the probability of a false-positive result was less than 0.2. In contrast, greater FPRP values were observed for the other significant associations between *CASP7* variants and gastric cancer, suggesting some possible bias in these findings.

**Table 6:** False-positive report probability values for associations between risk of gastric cancer and frequency of genotypes of the *CASP7* gene.

Genotype/Haplotype	Crude OR^a^ (95% CI)	*P* b	Statistical power^c^	Prior probability				
				0.25	0.1	0.01	0.001	0.0001
*CASP7* rs4353229								
TT vs. CC/CT	0.82 (0.69–0.97)	0.023	0.997	0.065	**0.172**	0.696	0.958	0.996
TT vs. CC/CT								
≤59	0.77 (0.60–0.98)	0.035	0.863	0.109	0.267	0.801	0.976	0.998
Never smoker	0.77 (0.61–0.91)	0.026	0.873	0.082	0.211	0.747	0.967	0.997
Never drinker	0.80 (0.65–0.98)	0.028	0.954	0.081	0.209	0.744	0.967	0.997
Non-cardia	0.80 (0.66–0.96)	0.019	0.964	0.056	**0.151**	0.661	0.952	0.995
Combined *CASP7* risk genotypes								
1–2 vs. 0	0.82 (0.68–0.99)	0.042	0.998	0.112	0.275	0.806	0.977	0.998
3–4 vs. 0	0.87 (0.75–1.00)	0.046	0.976	0.417	0.682	0.959	0.996	1.000
≥1 vs. 0								
≤59	0.75 (0.59–0.95)	0.019	0.807	0.066	**0.175**	0.700	0.959	0.996
Female	0.72 (0.53–0.99)	0.040	0.671	0.152	0.349	0.855	0.983	0.998
Never smoker	0.76 (0.60–0.96)	0.019	0.848	0.063	**0.168**	0.689	0.957	0.996
Never drinker	0.78 (0.64–0.96)	0.016	0.931	0.049	**0.134**	0.630	0.945	0.994
Non-cardia	0.78 (0.65–0.94)	0.010	0.943	0.031	**0.080**	0.512	0.914	0.991

a The crude OR reported in Table 2.

b The chi-square test of the genotype distributions reported in Table 2.

c Statistical power was calculated using the number of observations in the study and the OR and *P* values in this table.

**Figure 2:**
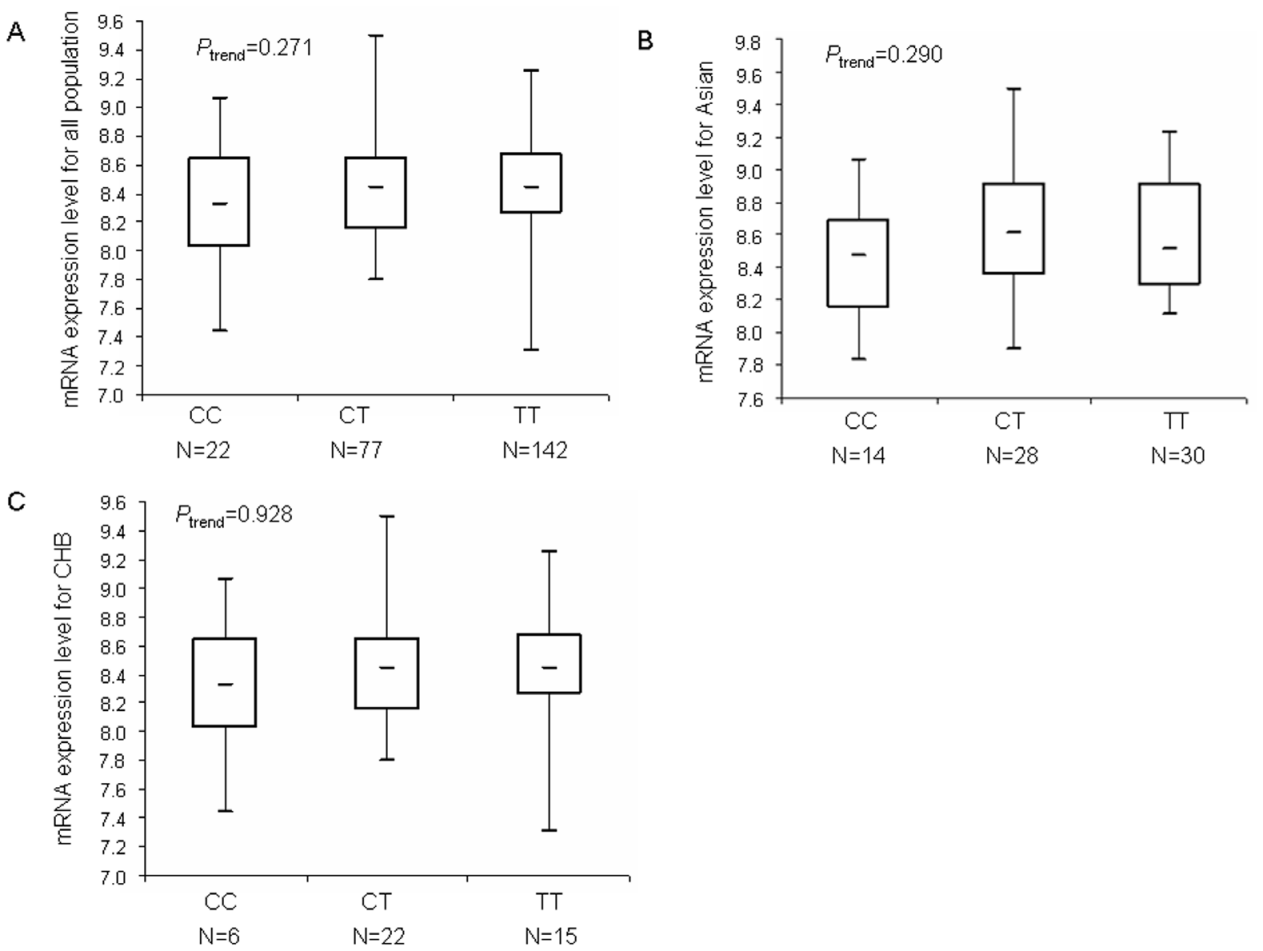
mRNA expression level of the *CASP7* gene in EBV-transformed lymphoblastoid cell lines. (A) mRNA expression in 261 HapMap cell lines of all population. (B) mRNA expression in 82 HapMap cell lines of unrelated Asian. (C) mRNA expression in 45 HapMap cell lines of unrelated CHB.

In addition, we also performed the genotype-phenotype correlation analysis for the *CASP7* rs4353229 T>C SNP by using the genotyping and mRNA expression data for CASP7 from the 270 lymphoblastoid cell lines available online (**Figure 2**). We found that the T allele, compared with the C allele, appeared to be correlated with high mRNA expression, consistent for Asians (n = 82), Chinese (n = 43) and all populations (n = 241), although none of the differences was statistically significant, likely due to small sample size.

## Discussion

In this hospital-based case-control study, we investigated the associations between four common, putatively functional SNPs located in the 3′ UTR of the *CASP7* gene and risk of gastric cancer in an Eastern Chinese population. When we evaluated each SNP separately, only the rs4353229 homozygous TT variant genotype was associated with significantly reduced gastric cancer risk in a recessive model, and this risk was more evident in subgroups of younger subjects, non-smokers, non-drinkers, and NGCA. In the combined analysis, we found that subjects with ≥1 *CASP7* risk genotypes had a significantly decreased risk of gastric cancer, and this risk was more evident in subgroups of younger subjects, females, non-smokers, non-drinkers and NGCA. However, it is obvious that rs4353229 T>C SNP contributed to most of the effect observed for the combined genotypes. Therefore, these findings suggest that the *CASP7* rs4353229 T>C SNP may cause susceptibility to gastric cancer and could be used as a biomarker for the disease. To the best of our knowledge, this is the first post-genome wide association studies (GWAS) study that investigated the association of putatively functional *CASP7* SNPs with gastric cancer risk.

Apoptosis is a mode of programmed cell death that provides a protective mechanism by removing DNA-damaged cells and thus plays a central role in maintaining homeostasis. [6] Alterations of apoptosis may contribute to the pathogenesis of a variety of human diseases, including cancer. Apoptosis depends on activation of caspases in response to diverse cell death stimuli that will then lead to proteolysis of numerous cellular proteins, resulting in the biochemical and morphological changes typical of this form of death [23]. Caspase-7 has been identified as one of the three executors of apoptosis in mammalian cells, which is crucial for the execution phase of apoptosis and contributes to mitochondrial events, such as the loss of mitochondrial membrane potential and the release of proapoptotic factors including cytochrome c and apoptosis-inducing factor [24], [25].

In a previous study, Soung et al. suggested that inactivating mutations of the *CASP7* gene, the 70 Cys to Tyr mutant, may lead to the loss of its apoptotic function and thus contribute to the pathogenesis of some human solid cancers [10]. An animal study revealed that caspase-7-deficient mice were resistant to lipopolysaccharide (LPS)-induced lymphocyte apoptosis and were markedly protected from the LPS-induced lethality [26]. In light of biological importance of the *CASP7* gene, it is biological plausible that genetic variations in *CASP7* could affect cancer risk through altering protein expression or functions of caspase-7. Indeed, in human studies, caspase-7 was also found to be down-regulated in gastric cancer [27] and colonic carcinoma [28].

A few previous pre-GWAS studies have investigated the associations between genetic variations in *CASP7* and cancer risk. Lee et al. examined four SNPs of *CASP7* in a case-control study that consisted of 720 Korean lung cancer patients and 720 healthy controls, and they found that only rs2227310 homozygous GG variant genotype was associated with a significantly increased risk of lung cancer, compared with the rs2227310 wild-type CC genotype [29]. Xu et al. found that five SNPs in *CASP7* were associated with the risk for endometrial cancer in a Chinese population [30]. In another Korean study conducted by Park et al., two SNPs of *CASP7* (rs12416109 and rs3814231) were found to be associated with risk of childhood leukemia [31]. Liu et al. also found rs3127075 in *CASP7* was associated with an increased risk of esophageal adenocarcinoma in 335 cases and 319 controls in Caucasian population [32]. Because most of the SNPs selected in the previous studies, including GWASs, were either tagging SNPs or SNPs located in exons, they were therefore not included in the present study.

A growing body of evidence indicates that miRNAs, which bind to the 3′UTR of mRNAs of their target genes, play an important role in carcinogenesis by regulating the expressions of proto-oncogenes or tumor suppressor genes [33]. SNPs located in the miRNA-binding site of a miRNA target are likely to disrupt miRNA–mRNA interaction, resulting in the deregulation of target gene expression [34]. In the present study, we selected four potentially functional SNPs located in the 3′UTR of *CASP7*, which were predicted by software SNPinfo as located in the miRNA binding sites. We found that rs10787498 homozygous GG variant genotype to be associated with non-significantly decreased risk of gastric cancer, which was consistent with the results in the study of endometrial cancer by Xu et al [30]. We did find, however, that the rs4353229 T>C was associated with decreased risk of gastric cancer under a recessive model.

We used publically available data on *CASP7* genotypes and transcript expression levels available online for the genotype–phenotype association analysis. It appeared that the rs4353229T allele was associated non-significantly increased levels of RNA expression for Asians, Chinese and all populations, which may be due to the relative small sample size. Therefore, additional larger studies with the target tissues and more mRNA samples or further functional studies of the rs4353229 C>T SNP are warranted to reveal the possible mechanisms underlying the observed associations. The rs4353229 SNP was in strong LD with the rs2227309 SNP in *CASP7* (r^2^ = 0.92; D’ = 0.97), which has been found to be associated with *CASP7* expression level and risk of rheumatoid arthritis by Garcia-Lozano et al [35]. However, it is also likely that the association between the rs4353229 genotypes and risk of gastric cancer may be due to its LD with other unknown nearby functional variant(s).

In stratification analysis, our data suggested that the observed risk was more pronounced in younger subjects, never smokers or never drinkers, because younger patients tended to be less exposed to smoking and drinking, as we also observed some protective effect in non-smokers and non-drinkers, who were likely to have been experienced some light exposures, if any. This is consistent with the notion that susceptible individuals are likely to be young with light exposure. In contrast, older subjects may have been exposed to more environmental hazards that may overcome their genetic advantage of carrying the protective alleles. A more pronounced protective effect was observed among women other than men, which may be partly explained by roles of different hormones or exposure patterns between sexes. However, these speculations need to be further substantiated in future large studies and should be treated with caution due to the limited sample size, particularly for the stratified analyses, in the present study.

Since cancers are complex diseases involving multiple genetic variations and gene–environment interactions, not a single locus can fully explain their genetic susceptibility. Recent advances in gene-gene interaction have been used in previous studies, such as a multi-analytic strategy combining logistic regression (LR), MDR and classification and regression tree (CART) approaches [36], [37]. In the present study, we used MDR to detect a high order of dimensions and characterize nonlinear interactions among genetic and environmental attributes. We found that the overall best MDR model was the eight factors model that includes all the four SNPs and the four exposure status, which indicates potential gene–environment interactions. Logistic regression was also applied to investigate multiplicative interactions between these four SNPs and exposures. But there was no statistical evidence for interactions between the variant genotypes and any of the tested exposure variables on risk of gastric cancer, which may be due to the relative small sample size in the subgroups. Larger studies are needed to further validate our results.

There are a number of possible limitations in the present study. First of all, this is a hospital-based case–control study with possible selection bias, because the cases recruited from a cancer hospital may not be ideal representatives of all cases originated from the geographically matched population with similar environmental exposure. However, potential confounding bias may be minimized by frequency-matching cases and controls on age, sex, areas of residence and further adjustment for potential confounding factors in final analyses. The genotype distribution for each SNP was in Hardy–Weinberg equilibrium, which further supports the randomness of our control selection. Secondly, only potentially functional SNPs, i.e., at the miRNA binding sites of *CASP7*, one gene among of many genes involved in the apoptosis pathway, were selected for investigation in the present study. Since cancer is a complex and multifactorial disease, any single SNP may not be sufficient enough for the prediction of the overall risk [38]. Future studies should include more SNPs of more genes involved in the apoptosis pathway to combine the modest effect of each SNP and enhance their predictive power. Finally, *H. pylori* infection, one of the major risk factors for gastric cancer, was not evaluated for patients included in the present study, because our hospital did not perform such detection for routine diagnosis. The joint effects of *H. pylori* and *CASP7* genotypes should be explored by additional prospective studies with well-designed clinical investigations to further confirm our findings.
